# Examining the quantum fisher information in the interaction of a dirac system with a squeezed generalized amplitude damping channel

**DOI:** 10.1038/s41598-024-76007-7

**Published:** 2024-10-18

**Authors:** C. Iyen, M. S. Liman, S. J. Emem-Obong, W. A. Yahya, C. A. Onate, B. J. Falaye

**Affiliations:** 1 Department of Physics, Federal University of Lafia, Lafia, Nigeria; 2 Department of Pure and Applied Physics, Federal University Wukari, Wukari, Nigeria; 3 Department of Physics and Materials Science, Kwara State University, Malete, Nigeria; 4 Department of Physical Sciences, Bowen University, Iwo, Nigeria; 5 Department of Physics, Anchor University, Ayobo, Lagos, Nigeria

**Keywords:** Quantum information, QFI, Decoherence, Metrology, Open system, Noisy channel, Information theory and computation, Quantum physics

## Abstract

The inherent association between real quantum systems and their surrounding environment invariably results in decoherence, leading to the loss of entanglement. This diminution in entanglement coincides with a decline in the fidelity of transmitted information using the entangled quantum resource. This study scrutinizes the impact of the squeezed generalized amplitude damping (SGAD) channel on quantum Fisher information (QFI) parameters. The SGAD channel model, a versatile framework, is also employed to simulate other dissipative channels, including amplitude damping (AD) and generalized amplitude damping (GAD). Kraus operators facilitate the modeling of noisy channels. The results reveal that, within the SGAD channel, the QFI remains impervious to the squeezing variables (r and $\Phi$). In the GAD channel, $F\theta _{GAD}$ undergoes enhancement to a constant value with an upswing in temperature (T), while the $\phi$ parameter in the GAD channel, $F\phi _{GAD}$, akin to the SGAD channel, surges around T = 2 before complete loss ensues. Concerning the AD channel, the $\theta$ component of the QFI initially experiences decoherence with an augmentation in the AD noise parameter ($\lambda$). Subsequently, it is restored to its initial value with a further escalation in $\lambda$. Conversely, the $\phi$ component of the QFI in the AD channel experiences decoherence with an elevation in the AD noise parameter ($\lambda$).

## Introduction

The need for constant miniaturization of electronic circuits^1,2^, increase in problem solving speed^3,4^, development of unbreakable codes^5,6^, and secure communication^7,8^ has made quantum information an exciting and highly researched area of science and technology^9–11^. Despite its promising advantages, quantum information is confronted with numerous challenges, including quantum decoherence^12,13^, error correction^14–17^, hardware development^18–20^, and quantum measurement^21–23^, among others. Of these challenges, quantum decoherence and quantum measurement raise serious concerns. Quantum decoherence, in particular, poses a significant threat as it directly impacts the primary quantum resource exploited for quantum information, namely, quantum entanglement.

When particles are seperated by a distance, they exhibit a peculiar connection, that seem unaffected by the actual physical distance between them. This phenomenon is attributed to entanglement, where actions performed on one entangled particle instantaneously influence others within the entangled group^24–28^. In realistic quantum scenarios, open quantum structures are crucial considerations. Open quantum systems, characterized by interactions and susceptibility to environmental conditions, are integral due to their impact on entanglement. The interactions of open quantum systems with their surroundings, however, introduce adverse effects, leading to a phenomenon known as decoherence. Decoherence, defined as the loss of coherence or entanglement, results in the loss of information^29–31^.

To investigate the effect of the environment on quantum information, it is necessary to measure the original parameters of the quantum system both before and after it encounters the noisy channel. . The measurement of quantum parameters is known as quantum metrology. The goal of quantum metrology is to obtain precise measurements by utilizing quantum concepts^32,33^. There are several methods of quantum metrology, namely Quantum Cramer-Rao Bound, QFI, Heisenberg-Limit Metrology, Time-Evolution-Based Metrology, and lots more. However, QFI provides a universal metric for quantifying the precision of parameter estimation in quantum systems, which makes it a versatile tool for assessing the performance of different metrology techniques.

QFI is a pivotal metric in quantum metrology^34^ quantifying the ultimate precision attainable in estimating parameters encoded within quantum states. While extensive research has illuminated QFI without considering the effect of noisy channels on its comprising parameters^35,36^, the formidable influence of dissipative processes, which are ubiquitous in practical quantum environments, remains a compelling frontier. Dissipative channels, characterized by their capacity to induce decoherence and information loss, have emerged as a critical area of study^37,38^. In this context, Kraus operators^39^ provide a powerful mathematical foundation for describing the behaviors of open quantum arrangements. They serve as the linchpin for understanding how dissipative interactions transform quantum states, thus mediating the behavior of QFI.

Within the family of dissipative channels, the amplitude damping (AD) channel and its generalizations, notably the SGAD channel, are particularly significant. These channels encapsulate a range of physical scenarios, from energy dissipation to more complex interactions with external environments. Understanding the effect of these channels on the quantum Fisher information (QFI) is vital for optimizing the use of quantum resources in realistic environments. Several studies have investigated the behavior of QFI in noisy channels. For example, Falaye *et al.*^39^ examined how the QFI of an N-qubit Greenberger-Horne-Zeilinger (GHZ) state is affected by exposure to decoherence channels. They focused on the bit-phase flip (BPF) and generalized amplitude damping (GAD) channels, which are experimentally realizable.

The eigenvalues and the QFI were computed after determining the changes induced by these channels. It was found that, in the absence of environmental interactions, the Heisenberg limit can be achieved through z-direction rotary motions. Moreover, they observed that the maximum average QFI of the N-qubit GHZ state declines in the BPF channel as the decoherence rate $\rho _B$ increases, due to information leakage from the system to the environment^39^. This decline continues until $\rho _B$ reaches 0.5, after which the QFI recovers, forming a symmetrical pattern around $\rho _B = 0.5$. Notably, when $\rho _B > 0.5$, the noise paradoxically leads to greater efficiency. Additionally, their study revealed that QFI decays more rapidly in the GAD channel with rising temperature. However, they also discovered that adjusting the environmental temperature could enhance the QFI.

Metwally and Ebrahim^40^ investigated a two-qubit system moving with increasing speed initially created in a full or partial condition of entanglement that has a local association with white-color sources of noise. They observed that, because of the changes in speed in the system as well as the impact of the noise, the entanglement deteriorated. Consequently, they used the concurrence to study the impact of noise magnitude, beginning state configurations, and acceleration on the survival of entanglement. The beginning variables used to characterize their arrangement were determined by making use of the QFI, in which two varieties were taken into account, which are single as well as double qubit varieties. Their findings demonstrated that the estimation degree of these variables obtained with the double qubit arrangement is higher than that obtained with the single qubit arrangement. As far as we are aware, no published research currently exists on how dissipatively noisy channels-namely, AD, GAD, and SGAD-affect an open Dirac system’s QFI parameters.

Reference^41^ worked on enhancing the teleportation of quantum Fisher information under the correlated generalized amplitude damping (CGAD) noise, they found out that the CGAD noise can enhance the teleported QFI, they also introduced a probabilistic scheme to improve the teleportation of QFI in CGAD noise by weak measurement (WM) and environment assisted measurement (EAM). They concluded that the EAM scheme is superior to the WM scheme with respect to QFI improvement under CGAD noise.

Reference^42^ investigated the bidirectional quantum teleportation of single-qubit state, for the case in which qubits fro m the quantum channels of teleportation are distributed by correlated noisy channels such as bit-flip, phase flip, depolarizing and amplitude damping. They found out that the existence of the noisy channel correlations reduced the effect of noise on the QFI.

In this research, we embark on a systematic exploration of QFI within the intricate landscape of dissipative noisy channels. Central to our analysis is the characterization of Kraus operators, which afford a fine-grained description of the dynamical evolution induced by dissipative processes. We probe the effects of AD, GAD, and SGAD channels, shedding light on their distinctive impacts on QFI. Kraus operators have been utilized to represent the effects of the channel’s noise. By elucidating the nuanced relationship between QFI and dissipative noisy channels, this work not only advances our theoretical understanding but also paves the way for practical applications in quantum technology. Harnessing the potential of dissipative resources for quantum-enhanced sensing, communication, and computation is an exciting prospect that beckons on the horizon.

The structure of this paper consists of the following: Section 2 delves into the subject of QFI; results are provided in Section 3, while the article is summarized in Section 4.

## Quantum fisher information

A notion from the study of exact measurement utilizing quantum systems, known as quantum metrology, is called QFI. It measures the amount of information a quantum state has about a certain parameter, such as the phase of a quantum wavefunction. Alternatively, it assesses how responsive a quantum state is to changes in a parameter. The fundamental idea of QFI is that a quantum state $\rho _\lambda$ is dependent on the values of unknown variables $\lambda$. The QFI may be expressed as Eq. (1) using the spectrum decomposition $\rho _\lambda =\sum _{i=1}^Np_i|\psi _i\rangle \langle \psi _i|$. Here, $\{|\psi _i\rangle \}$ establishes an orthogonal as well as full basis, and $p_i$ is the magnitude of $|\psi _i\rangle$.

1 $$\begin{aligned} F_\lambda =\underbrace{\sum _{i=1}^M\frac{1}{p_i}\left( \frac{\partial p_i}{\partial \lambda }\right) ^2}_{(I)} + \underbrace{\sum _{i=1}^M p_iF_{\lambda ,i}}_{(II)} -\underbrace{\sum _{i\ne j}^M\frac{8p_ip_j}{p_i + p_j}\biggl |\langle \psi _i|\frac{\partial \psi _i}{\partial \lambda }\biggr \rangle \biggr |^2}_{(III)}, \end{aligned}$$

where $F_{\lambda ,i}$ is as presented in Eq. (2).

2 $$\begin{aligned} F_{\lambda ,i}=4\left( \bigg \langle \frac{\partial \psi _i}{\partial \lambda }\bigg |\frac{\partial \psi _i}{\partial \lambda }\bigg \rangle -\bigg \langle \psi _i\bigg |\frac{\partial \psi _i}{\partial \lambda }\bigg \rangle \bigg |\right) . \end{aligned}$$

In the above expression, the first part (I) presents classical Fisher information, the second part (II) computes the QFI of the pure state, and the last part of the equation represents a mixed state contribution.

## QFI in noisy environment

This section delves into the interplay between QFI and dissipative noisy channels, focusing on the AD, GAD, and SGAD channels. It aims to uncover the complex dynamics that influence high-precision measurements in practical quantum systems, offering insights into how these channels impact the behavior and optimization of QFI in various quantum environments.

### QFI in SGAD channel

An SGAD channel^43^ is a quantum channel model that integrates the effects of both squeezing and amplitude damping. Squeezing is a quantum optical process that reduces noise in one quadrature of a light field while increasing noise in the conjugate quadrature. Amplitude damping, on the other hand, describes energy dissipation from a quantum system into its environment. The SGAD channel combines these effects by compressing the transmitted state and subjecting it to a finite-temperature bath.

Squeezing^44,45^ is a quantum operation that modifies a quantum state, reducing uncertainty in one observable (e.g., position) while increasing it in its conjugate observable (e.g., momentum). This operation is essential in quantum information processing and quantum optics. The GAD channel, which characterizes the interaction between a quantum system and its surroundings, is a generalization of the amplitude damping (AD) channel. In this model, the quantum system can lose energy, leading to changes in its state. The term “generalized” reflects its extension of the standard AD channel by accounting for additional phenomena, such as partial reflection of energy.

Now, when squeezing and a GAD channel are combined, it generates a channel that acts on quantum states in a way that incorporates both the effects of squeezing and the energy loss associated with the damping process. In practical terms, an SGAD channel would be a mathematical description of how a specific quantum system, which may be initially squeezed, interacts with its environment. This leads to both changes in its energy content and modifications in its squeezing parameters.

**Fig. 1:**
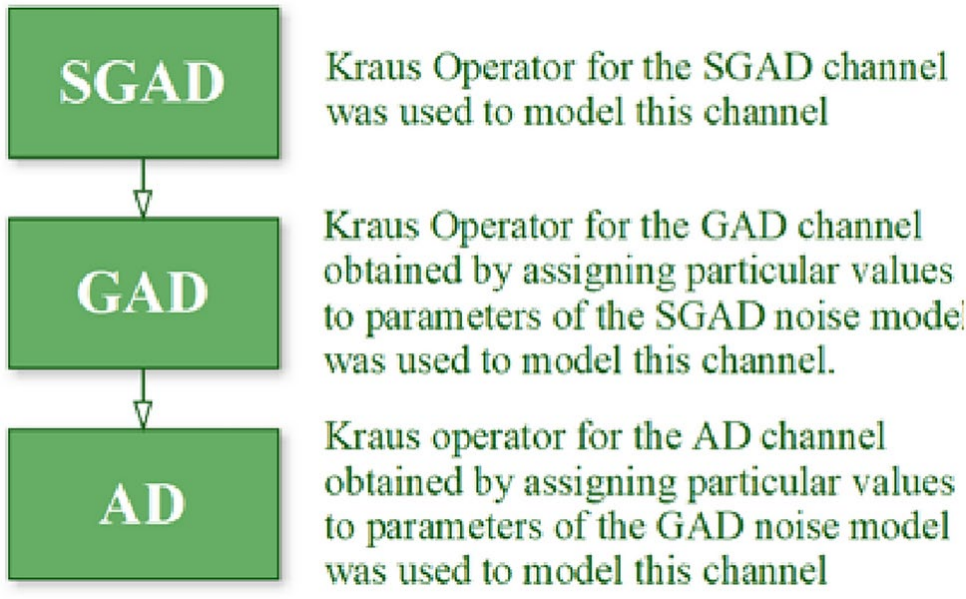
The flow of the research.

This type of channel is particularly relevant in scenarios where precision measurements are being performed on a quantum system that is subject to energy loss and decoherence due to its interaction with the surrounding environment. Understanding the behavior of such channels is crucial for designing robust quantum metrology protocols in realistic, noisy environments. In this research we have considered the QFI of the SGAD noisy channels and other noisy channels that are subset of the SGAD channel namely the GAD and AD channels, the flow of the research is as shown in Fig. 1.

SGAD channel Kraus operator is represented by the Eq. (3)^46,47^:

3 $$\begin{aligned} {\left\{ \begin{array}{ll} E_0^{S}= \sqrt{Q} \begin{bmatrix} 1 & 0 \\ 0 & \sqrt{1-\lambda }\\ \end{bmatrix} \quad E_1^{S}= \sqrt{Q} \begin{bmatrix} 0 & \sqrt{\lambda } \\ 0 & 0\\ \end{bmatrix} E_2^{S}= \sqrt{1-Q} \begin{bmatrix} \sqrt{1-v} & 0 \\ 0 & \sqrt{1-\mu }\\ \end{bmatrix} \quad E_3^{S}= \sqrt{1-Q} \begin{bmatrix} 0 & \sqrt{\mu }e^{i\Phi } \\ \sqrt{v} & 0 \\ \end{bmatrix}, \quad \end{array}\right. } \end{aligned}$$

where the variables $\mu$, v, and $\lambda$ are as shown in Eqs. (4), (5), and (6), respectively:

4 $$\begin{aligned} \mu= & \frac{2N+1}{2N(1-Q)} \frac{\sinh ^2(\frac{\gamma _0 a}{2})}{\sinh ^2 (\gamma _0 \frac{2N+1}{2})} e^{\frac{-\gamma _0 (2N+1)}{2}}, \end{aligned}$$

5 $$\begin{aligned} v= & \frac{N}{(1-Q)(2N+1)}(1-e^{-\gamma _0 (2N+1)}), \end{aligned}$$

6 $$\begin{aligned} \lambda= & \frac{1}{Q}(1-(1-Q)(\mu +v)-e^{(-\gamma _0(2N+1))}. \end{aligned}$$

**Fig. 2:**
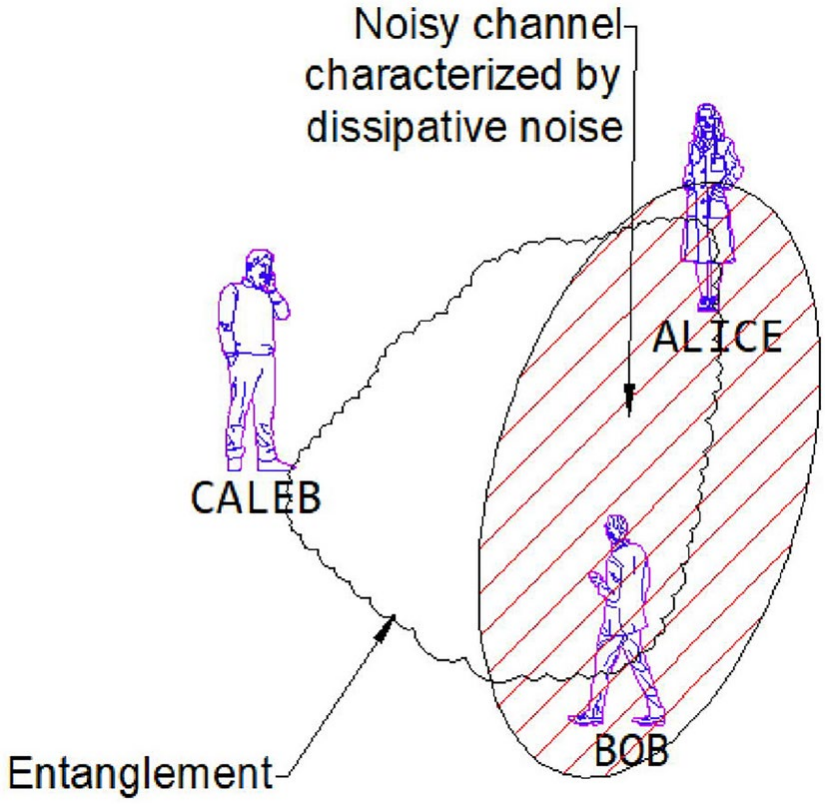
Illustration of a 3-qubit entangled state in which two of the qubits are subjected to a dissipatively noisy channel while the third qubit is not.

**Fig. 3:**
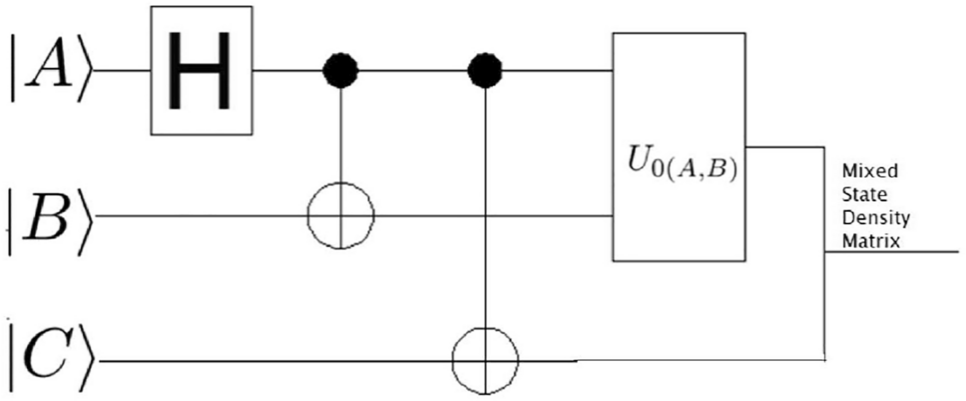
A quantum circuit illustration of a 3-qubit entangled state, where two of the qubits are exposed to a dissipative noisy channel, while the third qubit remains unaffected.

In the Eqs. (4), (5), and (6), $a=\sinh (2r)(2N_{th}+1)$, $N=N_{th}(\cosh ^2(r)+\sinh ^2(r))+\sinh ^2(r)$, $N_{th}=\frac{1}{e^{\left( \frac{\hbar \omega }{k_BT}\right) }-1}$.

In this research, we are only interested in the qualitative behaviour of the SGAD channel rather than its detailed time evolution; hence, the time parameter was ignored. We start by assuming that three individuals-Alice, Bob, and Caleb-are in possession of a quantum entanglement state, specifically a three-qubit entanglement state, and are situated at the same initial point in flat Minkowski space-time. Bob and Caleb operate detectors responsive to settings $|n\rangle _b$ and $|n\rangle _c$, respectively, while Alice’s detector can only detect mode $|n\rangle _a$ in the presence of decoherence. Considering that Alice, Bob, and Caleb are all in the asymptotically flat region, it becomes evident, as depicted in Fig. 2 and the corresponding quantum circuit in Fig. 3, that Alice and Bob are exposed to the SGAD Channel, while Caleb is not. The entanglement among Alice, Bob, and Caleb is represented by the following three-qubit initial entanglement state:

7 $$\begin{aligned} |\chi \rangle _{ABC}=cos(\theta )|0\rangle _A|0\rangle _B|0\rangle _C +sin(\theta )e^{i\phi }|1\rangle _A|1\rangle _B|1\rangle _C, \end{aligned}$$

here, $\theta$ and $\phi$ are known as the weight and phase parameters. To explore the QFI in dissipative channels, it is conventional to employ the Kraus operator for the SGAD channel-a comprehensive form encompassing both the AD and GAD channels. The Kraus operator for the SGAD channel is expressed in Eq. (3). The formulation for the physically accessible density matrix involving Alice, Bob, and Caleb within the SGAD channel can be derived by utilizing Eqs. (7) and (3).

8 $$\begin{aligned} \begin{aligned} \mathcal {K}^{SGAD}\rho _{ABC}=A^2\left( \left| 0_{\hat{a}},0_{\hat{b}},0_{\hat{c}}\right\rangle \right) \cdot \left\langle 0_{\hat{a}},0_{\hat{b}},0_{\hat{c}}\right| +AB\left( \left| 1_{\hat{a}},1_{\hat{b}},1_{\hat{c}}\right\rangle \right) \cdot \left\langle 0_{\hat{a}},0_{\hat{b}},0_{\hat{c}}\right| \\ +AB\left( \left| 0_{\hat{a}},0_{\hat{b}},0_{\hat{c}}\right\rangle \right) \cdot \left\langle 1_{\hat{a}},1_{\hat{b}},1_{\hat{c}}\right| +B^2\left( \left| 1_{\hat{a}},1_{\hat{b}},1_{\hat{c}}\right\rangle \right) \cdot \left\langle 1_{\hat{a}},1_{\hat{b}},1_{\hat{c}}\right| , \end{aligned} \end{aligned}$$

where $A=\cos (\theta )$ and $B=\exp (i \phi ) \sin (\theta )$. The eigenvalues of $\mathcal {K}^{SGAD}\rho _{ABC}$ are determined to be $e_1=B^2 \lambda Q \bar{\mu } \bar{Q}$, $e_2=Q^2 \left( B^2 \bar{\lambda }^2+A^2\right)$, $e_3=B^2 \lambda Q \left( \mu e^{2 \text {i}\Phi } \bar{Q}+\lambda Q\right)$, while $e_4=e_5=e_6=e_7=e_8=0$. The corresponding eigenfunctions are as follows:

9 $$\begin{aligned} |\Theta _1\rangle = \left( \begin{array}{c} 0 \\ 0 \\ 0 \\ 1 \\ 0 \\ 0 \\ 0 \\ 0 \\ \end{array} \right) , |\Theta _2\rangle =\left( \begin{array}{c} \frac{A}{B \sqrt{\left| \frac{A}{B \bar{\lambda }}\right| ^2+1} \bar{\lambda }} \\ 0 \\ 0 \\ 0 \\ 0 \\ 0 \\ 0 \\ \frac{1}{\sqrt{\left| \frac{A}{B \bar{\lambda }}\right| ^2+1}}, \\ \end{array} \right) |\Theta _3\rangle =\left( \begin{array}{c} 0 \\ 1 \\ 0 \\ 0 \\ 0 \\ 0 \\ 0 \\ 0 \\ \end{array} \right) . \end{aligned}$$

By employing Eq. (1) and recognizing that $\langle \psi _i|\frac{\partial \psi _i}{\partial \lambda }\rangle =0$, we obtain both the classical and quantum components of the QFI concerning $\theta$, which are then summed to yield:

10 $$\begin{aligned} \begin{aligned} |F\theta _{SGAD}\rangle&=\frac{4\bar{\lambda }^2 Q^2}{\bar{\lambda }^2 \sin ^2(\theta )+\cos ^2(\theta )}\\&+\frac{Q^2(2 \bar{\lambda }^2 \sin (\theta ) \cos (\theta )-2 \sin (\theta ) \cos (\theta ))^2}{\bar{\lambda }^2 \sin ^2(\theta )+\cos ^2(\theta )}\\&+4 \lambda Q \cos ^2(\theta )(\lambda Q+\mu \bar{Q} e^{2i\alpha })\\&+4 \lambda \bar{\mu } \bar{Q} Q \cos ^2(\theta ). \end{aligned} \end{aligned}$$

**Fig. 4:**
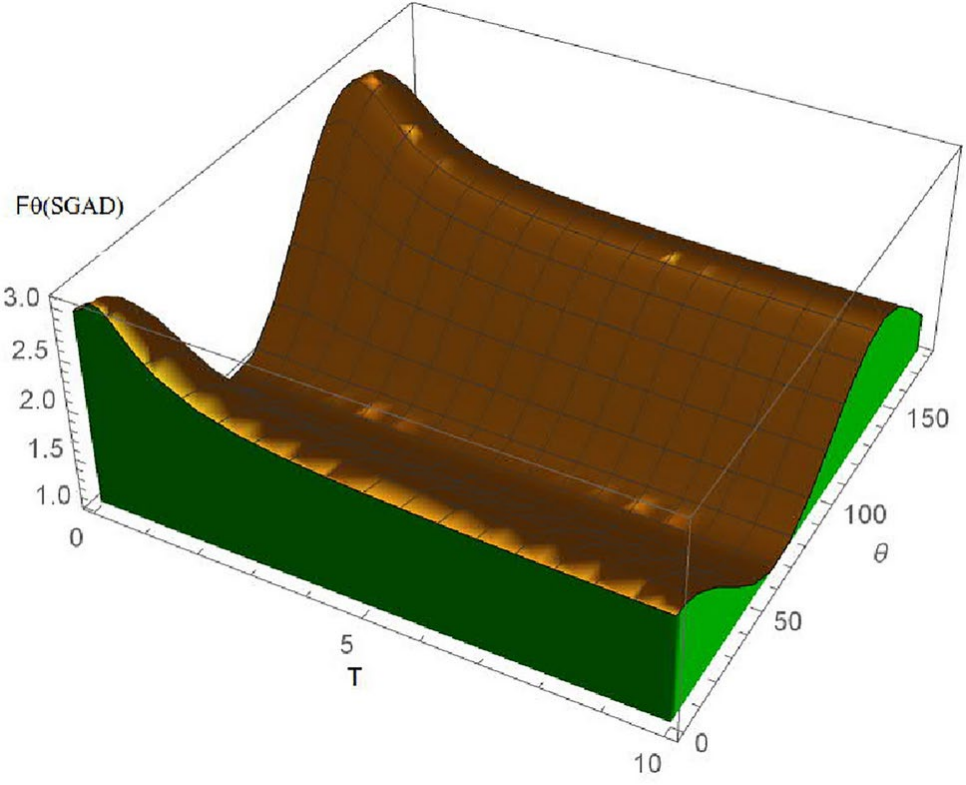
3D plot showing variation of $F\theta _{SGAD}$ with changes in the SGAD channel temperature T and the angle $\theta$.

**Fig. 5:**
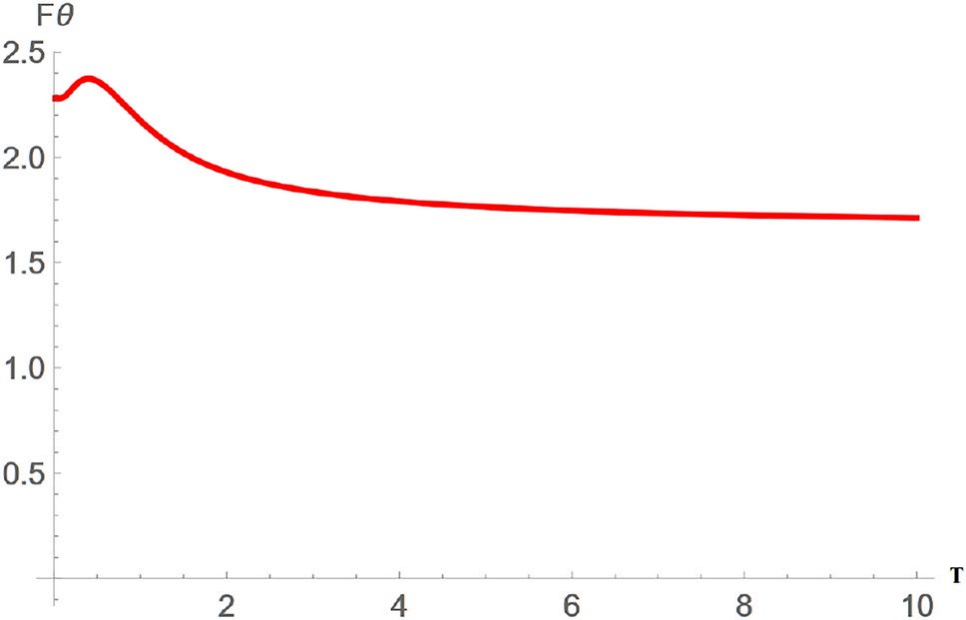
Plot showing variation of $F\theta _{SGAD}$ with changes in the SGAD channel temperature T.

From Eq. (10), it is evident that $F\theta _{SGAD}$ is independent of *v*, a component present in the Kraus operator for the SGAD channel. It is noted that *r* and $\Phi$ do not appear explicitly in the equation for $|F\theta _{SGAD}\rangle$. The characteristics of the SGAD channel are depicted in Figs. 4 and 5. As seen in Fig. 4, the QFI concerning $\theta$, denoted as $F\theta _{SGAD}$, is negatively influenced by an escalation in the channel temperature, but at higher temperature values, it is observed that the QFI for the $\theta$ parameter remains constant despite temperature increments. Additionally, it is noted that $F\theta _{SGAD}$ exhibits sinusoidal variations with alterations in the weight parameter $\theta$.

Figure 4 shows the variation of $F\theta _{SGAD}$ with variations in the channel temperature T and the weight parameters of the entangled particles $\theta$. It can be seen that $F\theta _{SGAD}$ declines with increasing temperature T which signifies an entanglement degradation with increase in T. This leads to a reduction in the sensitivity of the system to the weight parameter $\theta$ implying that the state is now in a mixed state and the quantum state has experienced decoherence which leads to a reduction in the fidelity of the information being transfered using the entanglement resource and an increased error rate. This decoherence is caused by thermal noise associated with the temperature of the channel T.

Figure 4 also shows the variation of $F\theta _{SGAD}$ with changes in $\theta$. The plot provides valuable insights into the precision and stability of the estimation process. High values of certain range of $\theta$ implies that $\theta$ can be estimated with high precision in that range, from Fig. 4, it is clear that $F\theta _{SGAD}$ has peaks at $\theta =0$ and $\theta =150$, this implies that $\theta$ can be measured with greater accuracy at those points. This is a particularly useful information in designing experiments and in quantum protocols in which precise control of $\theta$ is required, these are also the points in which the most precise measurements are made in quantum metrology. It is also clear from Fig. 4 that these peaks decline with increase in channel temperature T.

Figure 5 shows the variation of $F\theta _{SGAD}$ with changes in the SGAD channel temperature *T*. As shown in this figure, $F\theta _{SGAD}$ is adversely influenced by an intensification in the temperature, but at higher temperature range, it is observed that $F\theta _{SGAD}$ remains constant despite temperature increments. This illustrates the resilience of $F\theta _{SGAD}$ at high temperatures, providing a consistent measure for parameter estimation in the presence of noise. The initial slight surge in $F\theta _{SGAD}$ with increase in temperature might be attributed to the initial thermal fluctuations which might have introduced an initial correlation between particles. However, it is clear that $F\theta _{SGAD}$ declines to a constant value with increase in T, the effects of decline in $F\theta _{SGAD}$ has already been discussed earlier.

#### QFI in GAD channel

By designating particular dimensions for a subset of its variables, the SGAD can also be utilized to model the GAD channel. A quantum arrangement in a GAD channel releases and obtains kinetic energy through interactions with its surroundings. The GAD channel is required to reproduce the naturally occurring release of particles submerged in a vacuum bath at a temperature greater than zero. The Kraus operators for the GAD channel are provided by Eq. (11)^43^.

11 $$\begin{aligned} {\left\{ \begin{array}{ll} E_0^{G}= \sqrt{Q} \begin{bmatrix} 1 & 0 \\ 0 & \sqrt{1-\lambda _{G}}\\ \end{bmatrix} \quad E_1^{G}= \sqrt{Q} \begin{bmatrix} 0 & \sqrt{\lambda _{G}} \\ 0 & 0\\ \end{bmatrix} \quad \\ \\ E_2^{G}= \sqrt{1-Q} \begin{bmatrix} \sqrt{1-\lambda _{G}} & 0 \\ 0 & 1\\ \end{bmatrix} \quad E_3^{G}= \sqrt{1-Q} \begin{bmatrix} 0 & 0 \\ \sqrt{\lambda _{G}} & 0 \\ \end{bmatrix}. \quad \end{array}\right. } \end{aligned}$$

To obtain the GAD channel Kraus operators based on Eq. (11), we replace $\Phi = 0$, $\mu = 0$, and $v = \lambda$. Also, by using the equation for the QFI shown in Eq. (1) and the Kraus operators for the GAD channel represented in Eq. (11), the QFI in a GAD channel for both the classical and quantum parts is added together with respect to $\theta$ to obtain Eq. (12).

12 $$\begin{aligned} \begin{aligned} |{F\theta _{GAD}}\rangle =&4 \lambda ^2 Q^2 \cos ^2(\theta )+ \frac{4 (1-\lambda )^2 Q^2}{(1-\lambda )^2 \sin ^2(\theta )+\cos ^2(\theta )}\\&+\frac{Q^2 \left( 2 (1-\lambda )^2 \sin (\theta ) \cos (\theta )-2 \sin (\theta ) \cos (\theta )\right) ^2}{(1-\lambda )^2 \sin ^2(\theta )+\cos ^2(\theta )}+4 \lambda (1-Q) Q \cos ^2(\theta ). \end{aligned} \end{aligned}$$

Also, the classical and quantum parts with respect to $\phi$ are obtained as Eq. (13).

13 $$\begin{aligned} |F{\phi _{GAD} }\rangle =(1-\lambda )^2 \sin ^2(\theta )+\frac{4 (1-\lambda )^2 Q^2 \sin ^2(\theta ) \cos ^2(\theta )}{\cos ^2(\theta )}. \end{aligned}$$

**Fig. 6:**
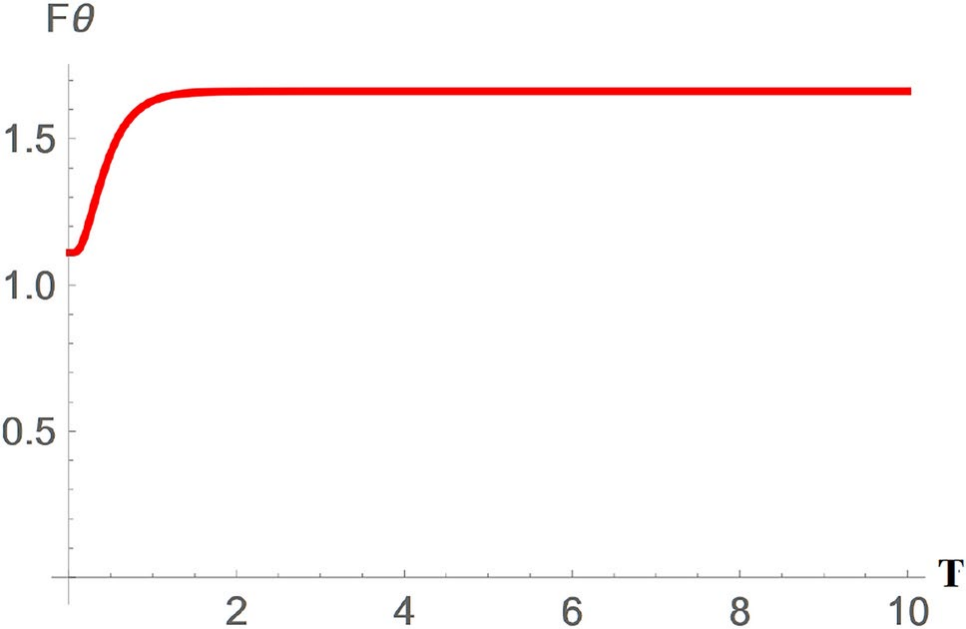
Plot showing variation of $F\theta _{GAD}$ with changes in the GAD channel temperature T.

**Fig. 7:**
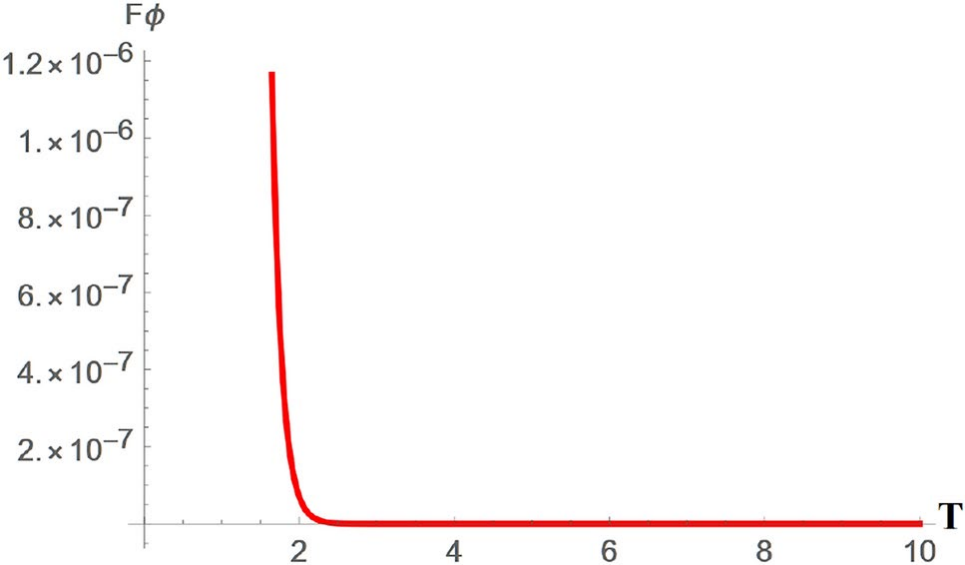
Plot showing variation of $F\phi _{GAD}$ with changes in the GAD channel temperature T.

**Fig. 8:**
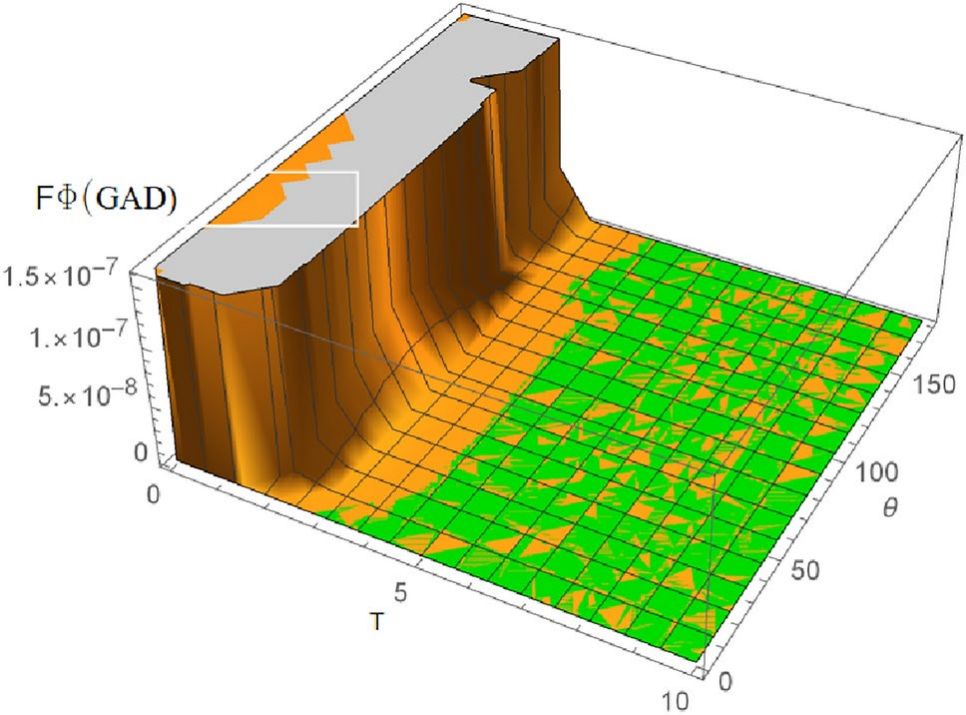
3D plot showing variation of $F\phi _{GAD}$ with changes in the GAD channel temperature T.

The characteristics of the QFI in the GAD channel are as shown in Figs. 6, 7, and 8. Figure 6 demonstrates the variation of the QFI $\theta$ parameter in a GAD channel ($F\theta _{GAD}$) changes in the channel temperature. Generally, from Fig. 6, it is evident that $F\theta _{GAD}$ initially increases with rising channel temperature, likely due to the same reasons discussed in Fig. 5. However, $F\theta _{GAD}$ reaches a peak value of approximately 1.65 as the temperature continues to rise. Given that similar assumptions were applied to both the GAD and SGAD plots, it is notable that $F\theta _{SGAD}$ attains a slightly higher peak of around 1.85, indicating that the inclusion of squeezing has further enhanced $F\theta _{SGAD}$ in comparison to $F\theta _{GAD}$.

Figures 7 and 8 show the variation of $F\phi _{GAD}$ with an increase in the GAD channel temperature T. From Fig. 7, it can be seen that $F\phi _{GAD}$ only has a spike at about when the channel temperature T is equal to 2, then drops immediately to 0 with increasing temperature. It is worthy of note that $\phi$ is a phase parameter. Gaps and spikes, as seen in the figures, are typically associated with phase transitions. From the plot, it can be deduced that there is the possibility of phase transitions with an increase in the channel temperature. Phase transitions are important in quantum communication protocols in which phase parameters are utilized, in associated error correction procedures, and in research focused on the dephasing nature of the SGAD channel.

#### AD channel

The AD channel is obtained from the GAD channel by equating $Q=0$. We use it to model a system that loses energy to the environment.The AD channel holds a foundational role in quantum information theory, serving as a vital element in the examination of quantum information systems. The AD channel, as a dissipative channel, operates by dissipating or transferring information from a quantum system to its environment. This process is accurately characterized by Kraus operators, which provide a set of transformations that outline the quantum state’s evolution during its interaction with the environment. Comprehending the dynamics of the AD channel proves crucial for managing noise and errors in quantum systems, establishing it as a pivotal element in the advancement of resilient quantum technologies. The Kraus operators for the AD channel are presented in Eq. (14)^43^.

14 $$\begin{aligned} {\left\{ \begin{array}{ll} E_0^{A}= \begin{bmatrix} 1 & 0 \\ 0 & \sqrt{1-\lambda _{A}}\\ \end{bmatrix} \quad E_1^{A}= \begin{bmatrix} 0 & \sqrt{\lambda _A} \\ 0 & 0\\ \end{bmatrix}. \quad \\ \\ \end{array}\right. } \end{aligned}$$

The rate of decoherence $\lambda _A$, which is such that $0\le \lambda \le 1$, indicates the possibility of an error occurring when particles travel through an AD noisy path. As stated previously, AD noise will affect only Alice’s and Bob’s qubits. For the AD channel, by following a similar procedure as in Section 3.2, the classical and quantum parts of the QFI in terms of $\theta$ were obtained, and after adding them up, Eq. (15) was obtained:

15 $$\begin{aligned} \begin{aligned} |{\hbox {F}\theta _{AD} }\rangle =&4 \lambda ^2 \cos ^2(\theta )+\frac{4 (1-\lambda )^2}{(1-\lambda )^2 \sin ^2(\theta )+\cos ^2(\theta )}\\&+\frac{\left( 2 (1-\lambda )^2 \sin (\theta ) \cos (\theta )-2 \sin (\theta ) \cos (\theta )\right) ^2}{(1-\lambda )^2 \sin ^2(\theta )+\cos ^2(\theta )}. \end{aligned} \end{aligned}$$

Also, the QFI in relation to $\phi$ is determined by the Eq. (16).

16 $$\begin{aligned} |F\phi _{AD}\rangle = \frac{(\lambda -1)^2 \sin ^2(2 \theta )}{(\lambda -1)^2 \sin ^2(\theta )+\cos ^2(\theta )}. \end{aligned}$$

**Fig. 9:**
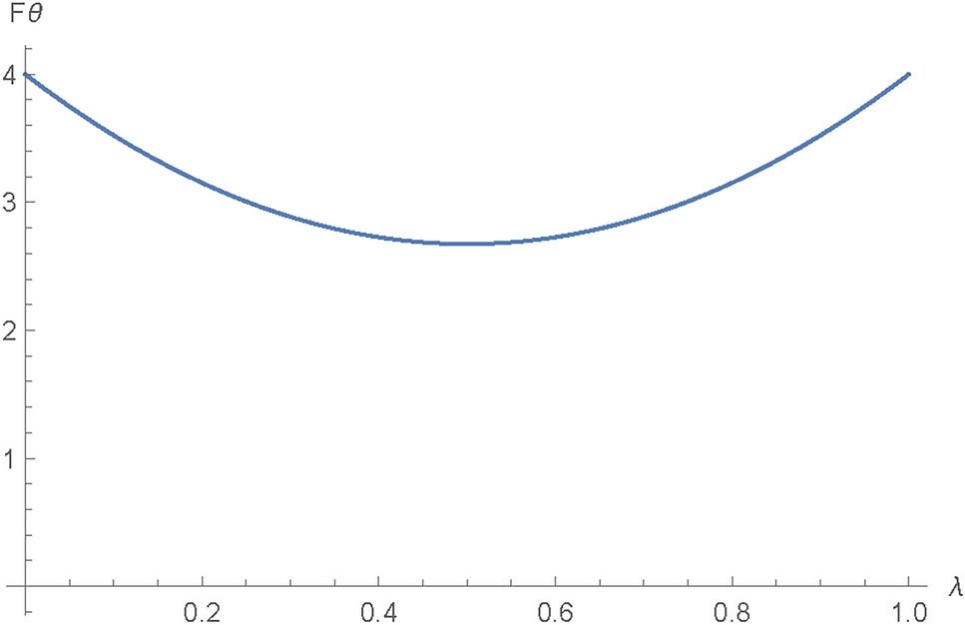
Plot showing variation of $F\theta _{AD}$ with changes in the AD channel noise parameter $\lambda$.

**Fig. 10:**
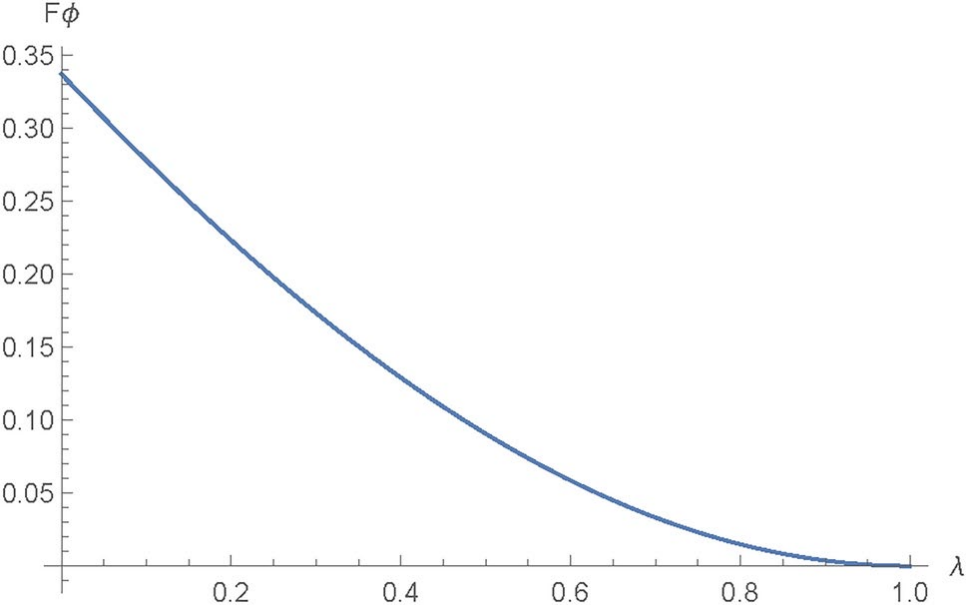
Plot showing variation of $F\phi _{AD}$ with changes in the AD channel noise parameter $\lambda$.

The attributes of the QFI in an AD channel are as shown in Figs. 9 and 10:

From Fig. 9, it can be seen that the $\theta$ attributes of the information initially decrease as the noise variable $\lambda$ for AD rises to a value of about 0.5, then it increases back to its initial value of 4.

Figure 10 shows how the $\phi$ parameter of the information being communicated, $F\phi _{AD}$, varies with changes in the AD noise parameter, $\lambda$. It can be inferred from the figure that the $\phi$ parameter of the information being transmitted diminishes with an increase in the AD noise parameter $\lambda$; the consequences of this decline have been extensively discussed earlier.

## Discussion and conclusion

In this study, we thoroughly examined the impact of dissipative noisy channels, particularly the amplitude damping (AD), generalized amplitude damping (GAD), and squeezed generalized amplitude damping (SGAD) channels, on the quantum Fisher information (QFI) of Dirac particles. The quantum system is represented by the entangled state $|\chi \rangle _{ABC}= \cos (\theta )|0\rangle _A|0\rangle _B|0\rangle _C+\sin (\theta )e^{i\phi }|1\rangle _A|1\rangle _B|1\rangle _C$. For the AD channel, where Alice and Bob are subjected to the noisy environment while Caleb is isolated from it, we observed noteworthy behaviors. Initially, the QFI related to the $\theta$ parameter, denoted as $F\theta _{AD}$, exhibits decoherence as the noise parameter $\lambda$ increases. However, beyond a critical threshold, $F\theta _{AD}$ recovers and returns to its initial value, producing a non-linear curve as $\lambda$ increases. At the same time, $F\phi _{AD}$, which pertains to the phase parameter $\phi$, experiences a steady damping with increasing $\lambda$.

In the case of the GAD channel, we observed that $F\theta _{GAD}$ is enhanced to a constant value as the channel temperature *T* increases, indicating a stabilization effect at higher temperatures. On the other hand, $F\phi _{GAD}$ displays a sharp spike at a specific temperature, which is dependent on the angle $\theta$. These spikes and discontinuities are indicative of phase transitions, suggesting that the system undergoes such transitions as the channel temperature *T* rises. These phase transitions are crucial in the implementation of quantum communication protocols, particularly those relying on phase parameters. Notable examples include the BB84 protocol with phase encoding^48,49^ and phase-based quantum bit commitment^50,51^ in quantum cryptography, as well as error correction procedures like phase correction in quantum teleportation.

Interestingly, the QFI parameters in the SGAD channel remain largely unaffected by the squeezing factors *r* and $\Phi$, suggesting that the channel maintains stability despite these variations. However, we found that $F\theta _{SGAD}$ decreases as the temperature *T* increases, indicating a reduction in entanglement, which in turn diminishes the fidelity of information transfer using the entanglement resource. This decline reflects the adverse effects of temperature on the precision of quantum information processing.

Further analysis reveals that the QFI for $\theta$ in the SGAD channel can be measured with maximum precision at $\theta = 0^\circ$ and $\theta = 150^\circ$, where the QFI reaches its peak values. However, these peaks tend to shift with increasing temperature, implying that the accuracy of quantum metrology is negatively influenced by higher channel temperatures. This demonstrates the nuanced interaction between quantum parameters and environmental conditions in dissipative channels.

A comparison with previous works underscores the unique contributions of this study. Ref.^52^ examines the impact of temperature and magnetic fields on quantum correlations in hybrid qubit-qutrit systems. In alignment with their findings, we observed that temperature has a detrimental effect on QFI, leading to decoherence, although in our case, the stability in the SGAD channel is notable under certain conditions. In contrast to Ref.^52^, where magnetic fields showed a variety of dynamical behaviors, our study focuses on the temperature’s role in QFI stability across multiple channels.

Moreover, Ref.^53^ investigates quantum correlations in a hybrid channel, revealing that combining different components can preserve quantum correlations even under local dephasing. While our study does not employ a hybrid channel, we similarly demonstrate that squeezing in the SGAD channel can help stabilize QFI against temperature fluctuations, aligning with the notion that certain configurations can lead to enhanced correlation preservation.

In Ref.^54^, the susceptibility of qubit entanglement to rapid decay under simultaneous global and local noise was highlighted. Our results, particularly regarding the GAD and SGAD channels, further extend this observation by showcasing how squeezing impacts coherence. Unlike the rapid decay observed in Ref.^54^, our work indicates that the SGAD channel preserves QFI over a wider temperature range, emphasizing the stabilizing effects of the channel design.

Finally, Ref.^55^ explores the quantum correlations in channels with symmetrical characteristics. Their findings on optimal parameterization for preserving entanglement in a hybrid channel resonate with our discovery of phase transitions in the GAD channel, where temperature plays a critical role. These transitions suggest that similar hybrid or composite channel designs could enhance quantum coherence and entanglement preservation.

Our findings contribute to the understanding of the complex dynamics within dissipative quantum channels and offer valuable insights into the manipulation of quantum resources in practical scenarios. The results align with previous research, which suggests that squeezing typically does not impair entanglement and may even enhance it, thereby reducing decoherence and improving the efficiency of quantum communication^46,56–58^.

This research not only provides a deeper understanding of the behavior of QFI in various noisy channels but also highlights the importance of considering environmental factors such as temperature when optimizing quantum systems for high-precision tasks.

## Data Availability

We do not have any research data outside the submitted manuscript file.
